# Repair of radiation damage to DNA

**DOI:** 10.1038/sj.bjc.6601729

**Published:** 2004-03-16

**Authors:** H Willers, J Dahm-Daphi, S N Powell

**Affiliations:** 1 Department of Radiation Oncology, Massachusetts General Hospital and Harvard Medical School, 100 Blossom Street, Boston, MA 02114, USA; 2 Department of Radiotherapy and Radiooncology, University Hospital Hamburg-Eppendorf, Martinistrasse 52, Hamburg 20246, Germany

**Keywords:** ionising radiation, DNA double-strand breaks, repair, recombination, replication

## Abstract

DNA double-strand breaks constitute the most dangerous type of DNA damage induced by ionising radiation (IR). Accordingly, the resistance of cells to IR is modulated by three intimately related cellular processes: DNA repair, recombination, and replication. Significant discoveries in this field of research have been made over the last few years. A picture seems to be emerging in which perturbations of recombination in cancer cells are a more widespread cause of genomic instability than previously appreciated. Conversely, such cells may also be more sensitive to certain chemotherapeutic drugs and to IR. Thus, the alterations in recombination that promote carcinogenesis by causing genomic instability may also be the weakness of the tumours that arise in this setting, a concept which could hold great promise for the advancement of cancer treatment in the not too distant future.

Exposure of the cellular DNA to ionising radiation (IR) inflicts various types of damage (Steel, 1996). It is established that the creation of a DNA double-strand break (DSB) represents the principal lesion that, if not adequately repaired, can lead to cell death via the generation of lethal chromosomal aberrations or the direct induction of apoptosis. Alternatively, an inaccurately repaired or unrepaired DSB may result in mutations or genomic rearrangements in a surviving cell, which in turn can lead to genomic instability and subsequently result in malignant cell transformation. Complex damage response pathways have evolved, and are evolutionary conserved, to protect the cell from the potentially deleterious effects of a DSB. Two principal recombinational repair pathways have been recognised, homologous recombination (HR) and nonhomologous end-joining (NHEJ), that employ entirely separate protein complexes. Briefly, DSB repair by HR requires an undamaged template molecule that contains a homologous DNA sequence, typically on the sister chromatid in the S and G2 phases of the cell cycle. In contrast, nonhomologous re-joining of two double-stranded DNA ends, which may occur in all cell-cycle phases, does not require an undamaged partner and does not rely on extensive homologies between the recombining ends. The study of these pathways has proved to be a rapidly evolving field of research over the past few years. Considerable interest has been generated by the realisation that defective HR and, in some cases, NHEJ can be causally linked to impaired DNA replication, genomic instability, human chromosomal instability syndromes, cancer development, or cellular hypersensitivity to DNA-damaging agents.

Here, we discuss some of the recent advancements in basic research on DNA repair, recombination, and replication, which could hold great promise for the advancement of cancer treatment in the not too distant future. To this end, the prevailing theme is that the genetic alterations in recombination that lead to genomic instability and malignant transformation may also determine how tumour cells respond to IR and certain chemotherapeutic drugs (Venkitaraman, 2003). We are only able to consider a small number of studies for the purposes of this mini-review. For further details on the molecular mechanisms and genetic determinants of HR and NHEJ, the reader is referred to recent review articles (Abraham, 2001; Khanna and Jackson, 2001; Pierce *et al*, 2001; Thompson and Schild, 2001; Jackson, 2002; Willers *et al*, 2002; Alberts, 2003; Powell and Kachnic, 2003).

## FROM REPLICATION TO RADIATION RESISTANCE

While exogenous DSBs are induced by IR or drugs such as bleomycin or etoposide, endogenous DSBs arise as byproducts of normal intracellular metabolism. It has been estimated that the spontaneous rate of endogenous DSBs may be as high as 50 breaks per cell per cell cycle (Vilenchik and Knudson, 2003). For example, DSBs can be detected when replication forks stall and collapse, a process that is thought to occur frequently during the S-phase. The cell can repair and/or restart replication forks by multiple mechanisms (Haber and Heyer, 2001), but the major mechanism that deals with replication-associated DSBs is HR. Intriguingly, replication intermediates can be deliberately broken or cleaved by the Mus81 endonuclease complex and the resulting DSB allows homology-mediated strand invasion, damage bypass, and reconstitution of the replication fork. The essential role of HR in replication is illustrated by the pronounced proliferative defect and embryonic lethality of mice with knockouts of genes that control HR, including the Rad51 recombinase or the breast cancer susceptibility genes BRCA2 or BRCA1 (Powell and Kachnic, 2003). Indeed, it has been suggested that the primary reason for the existence of HR is the maintenance of functional replication (Klein and Kreuzer, 2002). It is now clear that the cell takes advantage of the HR machinery to repair exogenous DSBs as well. Chromatid breaks in the S and G2 cell-cycle phases may be predominantly repaired by using the sister chromatid as a template. Therefore, genetic defects in HR can lead to both impaired DNA replication and enhanced IR sensitivity (Thompson and Schild, 2001; Powell and Kachnic, 2003). Moreover, impaired HR is also associated with, typically pronounced, hypersensitivity to DNA inter-strand crosslinks (ICLs), which result from cellular exposure to cytotoxic agents such as platinum compounds or mitomycin C.

However, the relationship between the control of replication and repair of IR-induced DSBs is likely not straightforward because DNA lesions caused by IR may not always be ideal substrates for repair by HR mechanisms that are normally employed during replication restart. IR typically generates clusters of ionisations, each containing at least 10 ionisations within a diameter of perhaps five or more nanometers (Steel, 1996). If such an event impinges on DNA, with the diameter of the double helix being approximately 2.5 nm, it would be expected to cause considerable local damage, including DSBs, single-strand breaks, and base damage. The repair of such a clustered damage site may be complex and/or slow and perhaps under a genetic control that has only partial overlap with the removal of endogenous DSBs. Indeed, mutations in genes that are involved in HR and replication often cause only modest or no radiation hypersensitivity. For example, the anti-recombinogenic effects of the BLM helicase, mutated in Bloom's Syndrome, or the p53 tumour suppressor are not thought to significantly modulate cellular radiation resistance (Dahm-Daphi, 2000; Thompson and Schild, 2001; Böhnke *et al*, 2004). Mutations in other genes that act in HR have been reported to increase radiation sensitivity by only ∼2-fold or less (Thompson and Schild, 2001). In general, mutations in NHEJ genes lead to greater radiation hypersensitivity than mutations in HR genes, suggesting that NHEJ is the dominant pathway for the removal of IR-induced DSBs. However, it is possible that this relationship changes when IR is combined with radiosensitising chemotherapy, which is the case in many cancer treatments.

Is there also a role for NHEJ in the repair of replication-associated DSBs? While it is difficult to envision how NHEJ should contribute to the restart of collapsed replication forks, NHEJ has been suggested to contribute to the repair of DSBs in the S-phase (Rothkamm *et al*, 2003). It is likely that the balance of NHEJ and HR in the removal of DSBs depends on the type and location of the lesion, among other factors. Of note, NHEJ is inherently error-prone and mutagenic, because this process is unable to faithfully restore the original DNA sequence, as opposed to HR, and because NHEJ itself can introduce sequence changes during repair. Still, NHEJ is suggested to be the dominant DSB repair pathway in mammalian cells, which is in part related to the fact that only a very small fraction of the genome is coding for genes and regulatory elements, and that small sequence changes are tolerated by the cells.

Replication, recombination, and repair are intricately linked and cannot be studied separately (Klein and Kreuzer, 2002; Alberts, 2003). It follows that it should be of crucial importance to understand the genetic determinants and molecular mechanisms of replication in tumour cells, in order to predict the effectiveness and outcome of cancer therapy. For example, the suggested primary role of the BRCA1/2-defined pathway is to facilitate HR in the bypass of stalled replication forks. As to the question of how cancer arises in cells with deficiencies in BRCA1 or BRCA2, it seems likely that the impaired function of HR is a key step, since this is the only established defect in BRCA2-deficient cells (Powell *et al*, 2002; Powell and Kachnic, 2003). A mutator phenotype can be triggered by a defect in the regulation of HR, either by making the process error-prone or by shunting repair events into a NHEJ pathway that is inherently mutagenic. Additional yet unknown mutational steps to bypass the proliferative block are also necessary so that net growth in the number of cancer cells can occur. It remains unsolved why this described defect in HR leads to the tissue-specific cancers of the breast and ovary. Proliferation in breast or ovarian epithelium may be associated with higher levels of endogenous DNA damage, relative to other tissues, which leads to replication stalling and requires HR. It is likely that defective HR in BRCA1/2-mutant tumour cells also underlies their hypersensitivity to IR and DNA crosslinking agents, which may ultimately impact on the chances of combination cancer therapy to achieve tumour eradication. However, to date there is no consistent evidence that BRCA1/2-mutant tumours carry a higher likelihood of radiocurability, but this is in large part due to the fact that residual tumour burden is the critical determinant of local control. Disentangling the complex relationship between tumour cell replication, genomic instability, cellular hypersensitivity to DNA-damaging agents, and clinical tumour control will be an area of great research interest in the years to come.

## DNA DAMAGE RESPONSE: 4D NOT 2D

**Figure 1:**
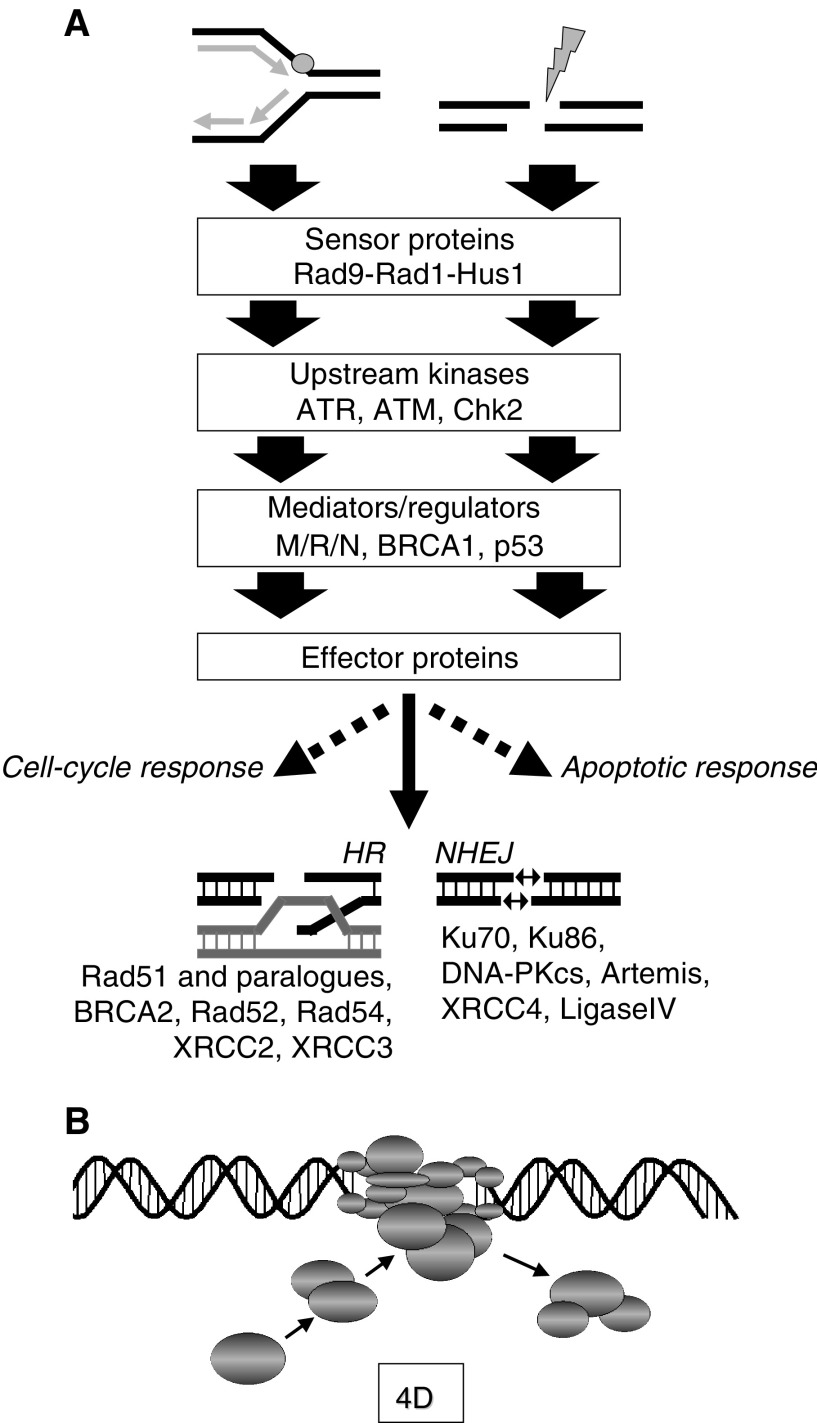
Principles of the cellular response to DSBs. (**A**) Replication arrest/damage or DSBs, such as induced by IR, are sensed by a set of proteins that include the ‘9–1–1’ complex (Rad9–Rad1–Hus1). The early responses kinases ATM, mainly acting on DSBs, and ATR, mainly acting on replication-associated damage, phosphorylate an extensive and partly overlapping spectrum of substrates. Upstream kinases and regulator/mediator proteins, in addition to including the Chk2 kinase, the Mre11/Rad50/Nbs1 complex (MRN), BRCA1 and p53 among others, affect recombinational repair, cell-cycle control, and stress and apoptotic responses. Repair processes by HR and NHEJ are genetically defined by distinct sets of effector protein complexes. (**B**) The damage response involves multiprotein complexes that are dynamic in chromosomal location and time (i.e., are regarded as four-dimensional entities).

Cells respond to exogenous and endogenous DSBs through a cascade of proteins ranging from sensors, which recognise the damage, through signal and mediator proteins to a series of downstream effectors that induce cell-cycle arrests, complete repair by homologous or nonhomologous mechanisms, or alternatively trigger cell death by apoptosis (for a review, Abraham, 2001; Jackson, 2002). A simplified illustration is shown in Figure 1A

. Defects at almost any step of this response pathway can result in measurable alterations of DNA repair by HR and/or NHEJ. Mutations upstream in the cascade, before the decision is made whether a lesion is repaired by HR or NHEJ, can directly affect both principal recombinational repair pathways. For example, mutation of the upstream kinase ATM, which is mutated in ataxia telangiectasia (AT), impairs HR and NHEJ (Luo *et al*, 1996; Khanna and Jackson, 2001), although it is yet unclear whether these alterations underlie the pronounced radiation hypersensitivity of AT cells. Loss of the Mre11 protein, which is mutant in the AT-like disorder (ATLD) and is usually found in a complex with Nbs1 and Rad50 (MRN complex), reduces both HR and NHEJ, which likely contributes to radiation hypersensitivity (Zhang *et al*, 2004; Zhang *et al*, unpublished). The MRN complex may be involved in the processing of DNA ends, among other functions, prior to the repair by HR or NHEJ. Both of the tumour suppressors p53 and BRCA1 control aspects of HR and NHEJ (Willers *et al*, 2000; Akyuz *et al*, 2002; Zhang *et al*, 2004; Willers *et al*, unpublished; Dahm-Daphi *et al*, unpublished), but the impact of these regulations on radiation resistance is a subject of active study. FANC-D2, which is mutated in a small subset of patients with the rare cancer predisposition and chromosomal instability syndrome Fanconi anaemia (FA), is likely implicated in HR via its protein interaction with BRCA2 (i.e., FANC-D1) (Howlett *et al*, 2002) and confers cellular radiation resistance – in contrast to most of the other genes in the FA pathway. Similarly, mutations of genes involved in execution of HR, such as BRCA2 or Rad51, compromise HR and radiation resistance, while mutations in the pathway controlled by DNA-PK affect radiation resistance via disruption of NHEJ. Increasing evidence points towards the existence of multiple subpathways of HR and NHEJ (Tutt *et al*, 2001; Akyuz *et al*, 2002; Jackson, 2002; Zhang *et al*, 2004), which will further complicate the understanding of the determinants of radiation resistance.

The illustration of the DNA damage response in a linear form (Figure 1A) should not be misleading. Several layers of complexity need to be considered. Response pathways do not represent linear sequences of events, but involve complex networks that include signaling cascades and feedback loops. The control of cell-cycle progression, DNA repair, and stress responses are not separate entities, but intricately linked. Thus, it is not surprising that many regulator and mediator proteins, such as BRCA1 or p53, appear to have pleiotropic functions in the response to genotoxic stress (Albrechtsen *et al*, 1999; Powell and Kachnic, 2003). Accordingly, they are components of multiple protein machines, which form at specific DNA damage sites. These machines represent sets of 10 or more spatially positioned interacting proteins that undergo highly ordered movements in a machine-like assembly (Figure 1B) (Alberts, 2003). When sets of proteins that can function in either replication, recombination, or repair processes assemble, it is crucial that their activities are highly regulated and applied only when and where they are needed. In other words, the multi-protein complexes that form in response to damaged DNA represent dynamic entities both in time and in subnuclear location. Examples include the BRCA1-associated genome surveillance complex (BASC), which contains in addition to BRCA1 proteins such as the BLM helicase and the MRN complex, but not BRCA2 (Wang *et al*, 2000), or the suggested dynamic formation of recombinosomes including Rad51, Rad52, and Rad54 at subnuclear damage sites (Essers *et al*, 2002). Therefore, the DNA damage response should be viewed as a four-dimensional system rather than as the linear, two-dimensional cascade that is typically drawn.

The concept of highly ordered and regulated protein machines at DNA damage sites may have profound implications for the interpretation of observed alterations in recombination-associated genes. Amino-acid changes in recombinational proteins have been increasingly reported in both sporadic tumours and in normal cells (Pierce *et al*, 2001; Mohrenweiser *et al*, 2003). Often, it is unclear whether such changes represent polymorphisms without functional consequences or (hypomorphic) mutations. Given the highly ordered nature of the described protein machines, small structural changes introduced by single amino-acid changes in individual proteins may already alter the activity of the complex significantly. Indeed, it appears that there is increasing evidence that mild reductions in DNA repair capacity, assumed to be the consequence of common genetic variation in the human population, affect cancer predisposition (Mohrenweiser *et al*, 2003), and, by inference, possibly modulate the response to radiation treatment as well.

## IMPAIRED RECOMBINATION: THE ACHILLES' HEEL OF CANCERS?

As we already discussed, mutations in the BRCA1–BRCA2–Rad51 pathway are associated with defective HR, and these may not only result in genomic instability but also determine the resistance of tumour cells to exogenous DSBs or ICLs. Conversely, inappropriate upregulation of HR activities could also contribute to genomic instability by causing homology-mediated aberrations such as certain chromosomal translocations or loss of heterozygosity. Some of these observations may be linked either to the observed overexpression of the Rad51 protein in several cell types or to the widespread disruption of pathways controlled by the p53 gene (Maacke *et al*, 2000; Linke *et al*, 2003), but it is yet unclear how these alterations affect radiation resistance (in the absence of apoptosis) (Albrechtsen *et al*, 1999; Dahm-Daphi, 2000; Böhnke *et al*, 2004). A direct clinical application of impaired HR control was suggested recently by the finding that the activity of the FA pathway, as determined by the methylation status of the FANC-F gene, dictated the sensitivity of several ovarian tumours to cisplatin (Taniguchi *et al*, 2003). Disruption of the pathway was found in 8–21% of tumours. An attractive hypothesis to be tested in clinical studies will be whether also methylation of BRCA1 in a significant fraction of sporadic breast and ovarian cancers, and possibly of FANC-F in breast tumours, can determine the response of such cancers to treatment with cross-linking drugs. Whether it will be possible to use the functional status of the FA pathway to predict the clinical tumour response to IR seems to be less clear.

In contrast to HR, there is as yet relatively little suggestion that implicates perturbed NHEJ pathways in the aetiology of genomic instability in solid cancers (Pierce *et al*, 2001; Jackson, 2002). In a mouse model, heterozygosity of DNA ligase IV, which is involved in NHEJ, promoted the development of soft-tissue sarcomas that possessed clonal amplifications, deletions, and translocations (Sharpless *et al*, 2001). Clinical correlation is still lacking, but in one recent report the SYT–SSX fusion gene controlled the expression of the NHEJ gene XRCC4 in a synovial sarcoma cell line (Xie *et al*, 2003). Interestingly, the cytotoxic agent Ecteinascidin 743 (ET-743), which has activity against soft tissue sarcomas, was found to exhibit increased toxicity in cells with defective NHEJ (Damia *et al*, 2001). It is tempting to speculate that, analogously to HR, both reduced or upregulated NHEJ can contribute to genomic instability at least in some tumour types and determine the cellular response to drugs that target NHEJ pathways.

**Figure 2:**
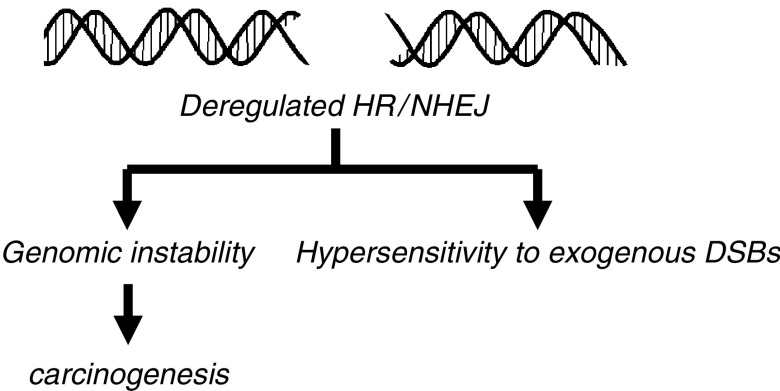
Aberrations in recombination and replication not only compromise genomic stability, thereby promoting the formation of cancers, but may also determine the sensitivity of tumour cells to treatment with DSB-inducing agents, including IR.

In summary, a picture is emerging in which perturbations of recombinational repair in cancer cells are a more widespread cause of genomic instability than previously appreciated. Conversely, such cells may also be more sensitive to certain chemotherapeutic drugs and IR. Thus, the alterations in recombination that promote carcinogenesis by causing genomic instability may also be the Achilles' heel of the cancers that arise in this setting (Figure 2

) (Venkitaraman, 2003).

## CONCEPTS OF THERAPEUTIC GAIN

Therapeutic gain is defined by a better relation between the killing of tumour and normal cells in a patient. The central goal of curative radiation therapy is to optimise therapeutic gain by maximising the likelihood of killing all clonogenic tumour cells, while keeping the damage to the surrounding normal tissues to a minimum. We distinguish between acute (or early) and late occurring normal tissue damage. Actively proliferating tissues such as mucosa typically express treatment-induced damage early. In contrast, in slow or nonproliferating tissues, such as the spinal cord or the kidneys, it can take many years before radiation damage becomes clinically manifest. While for the application of chemotherapy acute toxicity is typically dose-limiting, the total radiation dose that can be administered is typically limited by the development of late occurring normal tissue complications. Thus, in its simplest conception, targeting recombinational repair pathways could achieve therapeutic gain by the following mechanism: a novel combination of IR and a drug that disrupts HR pathways would preferentially kill proliferating tumour cells that are in S- or G2-phase and thus repair DSBs predominantly by interchromatid HR. Therapeutic gain will result, especially in relation to non- or slowly proliferating normal cells, which are largely in the G0- or G1-phase and repair mainly via NHEJ, because no sister chromatid is available. However, tumour cells that are quiescent and nonproliferating (such as hypoxic cells) may not be hit.

Alternatively, the presence of genetic mutations that shift DSB repair in cancer cells from NHEJ to the preferential use of HR pathways, including not only the inter-chromatid repair but also intra- or inter-chromosomal homology-mediated repair in G1, may confer particular susceptibility to novel drugs that disrupt HR. Such mutations could include the p53 gene, inactivation of which has been reported to result in elevated HR (Willers *et al*, 2000; Akyuz *et al*, 2002). In contrast, if mutations are already present in genes that promote HR, treatment with crosslinking agents such as cisplatin with or without IR should result in therapeutic gain.

Therefore, we envision that genotyping and/or phenotyping of individual cancers for DSB repair pathways could lead to a better prediction of how a tumour will respond to radiation therapy and certain chemotherapeutic agents that aim to generate lethal levels of DSBs in the target cells (Jackson, 2002). In addition, it may be necessary to analyse DSB repair in the normal tissues prior to therapy, because inherited alleles that contribute to genomic instability and carcinogenesis might also result in cellular radiation hypersensitivity. However, to date, inherited mutant alleles are usually present only in the heterozygous state, and loss of heterozygosity to homozygous deficient is only found in tumour cells.

## CONCLUSIONS: THE ‘R'S’ OF RADIATION RESISTANCE

Here, we have focused on some of the concepts and principles in the study of DNA replication, recombination, and repair (the three ‘R's’). These processes, which can no longer be considered separately, form a new paradigm for the understanding of the cellular resistance to radiation treatment. Genetic mutations in recombinational processes that affect replication and DSB repair may not only promote genomic instability but also determine the response of tumours to combination therapies with DNA damaging agents. This concept may provide the framework for future pre-clinical and clinical studies that discover and test novel combination therapies and tailor these to individual tumours. While some of our considerations are speculative at the present time, we anticipate that the rapid progress in this exciting field of research will continue over the next few years and provide many of the answers. Finally, although the focus of this review has been the contribution of cellular radiation resistance to the clinical tumour response, it is clear that additional factors, such as the contribution of the tumour microenvironment, are at least equally important in determining the likelihood of achieving tumour control and cure.
